# Examining respect, autonomy, and mistreatment in childbirth in the US: do provider type and place of birth matter?

**DOI:** 10.1186/s12978-023-01584-1

**Published:** 2023-05-01

**Authors:** P. Mimi Niles, Monique Baumont, Nisha Malhotra, Kathrin Stoll, Nan Strauss, Audrey Lyndon, Saraswathi Vedam

**Affiliations:** 1 New York University, 433 First Avenue, Room 644, New York, NY 10010 USA; 2 Every Mother Counts, 333 Hudson St Suite 1006, New York, NY 10013 USA; 3 University of British Columbia, BC Women’s Hospital, Shaughnessy Building E416 4500 Oak Street, Vancouver, BC V6H 3N1 Canada; 4 Department of Family Practice, Faculty of Medicine, University of British Columbia, Suite 320-5950 University Boulevard, Vancouver, BC V6T 1Z3 Canada

**Keywords:** Midwifery, Autonomy, Respectful maternity care, Homebirth, Birth center, Childbirth, Mistreatment

## Abstract

**Background:**

Analyses of factors that determine quality of perinatal care consistently rely on clinical markers, while failing to assess experiential outcomes. Understanding how model of care and birth setting influence experiences of respect, autonomy, and decision making, is essential for comprehensive assessment of quality.

**Methods:**

We examined responses (n = 1771) to an online cross-sectional national survey capturing experiences of perinatal care in the United States. We used validated patient-oriented measures and scales to assess four domains of experience: (1) decision-making, (2) respect, (3) mistreatment, and (4) time spent during visits. We categorized the provider type and birth setting into three groups: midwife at community birth, midwife at hospital-birth, and physician at hospital-birth. For each group, we used multivariate logistic regression, adjusted for demographic and clinical characteristics, to estimate the odds of experiential outcomes in all the four domains.

**Results:**

Compared to those cared for by physicians in hospitals, individuals cared for by midwives in community settings had more than five times the odds of experiencing higher autonomy (aOR: 5.22, 95% CI: 3.65–7.45), higher respect (aOR: 5.39, 95% CI: 3.72–7.82) and lower odds of mistreatment (aOR: 0.16, 95% CI: 0.10–0.26). We found significant differences across birth settings: participants cared for by midwives in the community settings had significantly better experiential outcomes than those in the hospital settings: high- autonomy (aOR: 2.97, 95% CI: 2.66–4.27), respect (aOR: 4.15, 95% CI: 2.81–6.14), mistreatment (aOR: 0.20, 95% CI: 0.11–0.34), time spent (aOR: 8.06, 95% CI: 4.26–15.28).

**Conclusion:**

Participants reported better experiential outcomes when cared for by midwives than by physicians. And for those receiving midwifery care, the quality of experiential outcomes was significantly higher in community settings than in hospital settings. Care settings matter and structures of hospital-based care may impair implementation of the person-centered midwifery care model.

## Introduction

Maternity care in the United States (US) is influenced by intersecting historical, political, economic, and social domains that converge to reproduce the current crisis of disparities. Equitable access to high quality care remains an elusive goal—as fragmented care, poor coordination, and a maldistribution of providers and care facilities perpetuate health inequities [1]. A recent report by the National Academies of Sciences, Engineering, and Medicine (NASEM) [1] demonstrates how the complex network of U.S. systems, institutional cultures, and policies impair access, collaboration, and coordination across provider types and birth settings.

The majority of births in the U.S. are attended by a physician (88%). The remaining 12% of births are attended by midwives. Of these midwifery-attended births, less than 2% occur in community settings (0.9% at home and 1.1% in freestanding birth centers [FBC]). Births occurring at home or in a FBC are termed ‘community birth’ throughout this paper [2]. Most (95%) of the U.S. midwifery workforce reports working exclusively in hospital settings [3]. The U.S. midwifery workforce is comprised of three different midwifery credentials –certified nurse-midwife (CNM), certified midwife (CM) and certified professional midwife (CPM). Most CNMs/CMs attend births in hospitals, with a smaller number providing care at homes and at birth centers. CNMs/CMs also provide primary care and gynecologic (which includes contraception and abortion). Scope of practice for CPMs, is in most cases limited to care during the perinatal cycle in community settings (homes- and birth centers). According to the most recent data on community births, nearly half (50.7%) of all home births were attended by CPMs and 30% were attended by CNM/CM midwives, with less than 1% attended by a physician. For birth center births, 56.6% of births were attended by CNM/CM midwives, 36.7% by CPM midwives, and only 2.7% by physicians [4]. Although largely distinct in educational and regulatory pathways, these midwifery credentials represent competencies and a shared commitment to protecting a person-centered, midwifery care model [5, 6].

Choice of provider and setting in the US maternity care system often requires navigating a complex network of barriers including insurance coverage, federal policies and state level regulations on both midwifery practice and community birth settings. While most pregnant people in the US give birth in hospital settings, there is significant variation in the availability of hospitals that provide perinatal care, types of maternity care providers, and access to community birth options. It is important to note that not everyone is able to access their preferred options. Compared with hospital births, people with planned home and birth center births are far more likely to have to pay for birth out-of-pocket rather than through public or private insurance coverage. For example, in more than half of all US states, over 70% of planned home births and 32% of birth center care were self-paid, while only 3.4% of hospital births were self-paid [4]. Though constituting a small share of overall births, the proportion of community births, attended by midwives, is gradually increasing. MacDorman et al. [7] reported that from 2019 to 2020, community births increased by 19.5%, birth center births increased by 13.2% and planned home births increased by 23.3%. Increases occurred in every U.S. state, and for all racial and ethnic groups, particularly non-Hispanic Black mothers (29.7%) [7].

In contrast to the standard obstetrical model, midwifery care is rooted in a philosophy that honors pregnancy and birth as a physiological, social and cultural process, not solely a clinical event [8–10]. The care relationship between the client and the midwife serves as the primary vehicle through which values such as autonomy, respect, and informed decision-making are operationalized to preserve an overall satisfying experience of childbearing [11, 12].

The physiologic benefits of community-based midwifery care are well documented [13–17]. In a Washington state cohort study, people who at the onset of labor planned to give birth outside of the hospital under the care of midwives experienced low cesarean birth rates of 11.4% in nulliparous individuals and 0.87% in multiparous individuals. The rates of adverse outcomes such as operative vaginal birth, episiotomy, fourth degree lacerations and other severe morbidities were also low [18]. Similarly, women receiving prenatal care at CMS-funded Strong Start birth centers experienced superior birth outcomes, regardless of whether they ultimately gave birth in the birth center or in a hospital compared to typical Medicaid care, even after adjusting for participant characteristics and medical and social risk factors commonly associated with poor birth outcomes [19].

Additional research confirms that experiential outcomes are enhanced when care is provided by midwives [20, 21]. A substantial body of evidence establishes the benefits of midwifery care [10, 22–24] but there is scarce research that quantifies experiential outcomes of care *across birth settings*. Experiential outcomes (such as trust, quality of relationship, decision making, autonomy, and respect) are consistently identified as top priorities by childbearing people [25, 26]. Individuals cared for by midwives report more respectful care, increased agency, and autonomy in decision-making compared to individuals cared for by physicians [27–29]. One study examining experiences of planned hospital births found that women receiving midwifery care had lower odds of holding back their questions; having a provider use words that were difficult to understand; and not feeling encouraged to discuss all of their concerns [30].

In a cross-sectional national survey in the US (n = 2700) [31] people cared for by midwives were much less likely to report mistreatment compared to those who had prenatal care from physicians. These authors also reported that compared to those who delivered in hospitals, those who planned community births reported fewer instances of mistreatment across racial groups. In a community setting, 6.6% of people of color reported mistreatment, compared to 33.9% of people of color who received care in a hospital [31].

The question remains, however, whether these differences persist within the same place setting, more specifically: Do experiential outcomes differ *in a hospital setting* among those cared for by physicians and hospital-based midwives? Our study further examines whether experiential outcomes of care by midwives vary based on the place setting, and identifies if experiential outcomes among those cared for by midwives in hospital-based settings differ from those cared for by midwives in community-based settings? We examined the differences across providers for across multiple experiential domains, which include: communication, autonomy, respect, mistreatment, and time spent with provider.

We acknowledge that pregnancy and birth are not limited to those who self-identify as “women,” or “mothers”. However, in Giving Voice to Mothers, a cross sectional survey, participants did not have the opportunity to identify their gender and the term ‘woman’ was used throughout the survey. The limited convention of offering a binary choice means we did not capture our participants gender identity. In this analysis, we use the terms ‘woman and childbearing person’, ‘participant’ and ‘person-centered’ care in our broader analysis and discussion, as our findings have implications for people across the gender spectrum with the capacity for pregnancy and birth.

## Methods

### Study design

We conducted a secondary analysis of data from the Giving Voice to Mothers US (GVtM) study. The cross-sectional survey was developed using a community-based participatory research process to document care experiences of people who were pregnant in the U.S. from 2010 to 2017. This study was designed to document the experiences of pregnant/parenting people of color and those who planned to give birth at home or in a freestanding birth center. The survey explored preferences for care, interactions with providers, experiences of respect and safety, autonomy in decision-making, and access to care options. The full GVtM survey instrument (218 items) was available in English and Spanish. Scale items had pre-defined Likert response options. Details on survey development and recruitment are available in a previous publication [31, 32]. A full report of the findings is also publicly available. The Behavioural Research Ethics Board at the University of British Columbia approved the study (H15-01524).

### Recruitment

Surveys of childbearing people’s experiences with pregnancy and birth care are often restricted to those giving birth in hospitals and overrepresent white participants [33]. To address this under-representation, people of color and people who planned a birth either at home or at a freestanding birthing center were oversampled. Invitations to participate were shared through NGO memberships and listservs, social media, doulas, midwives, physicians, and at professional conferences. Recruitment was also embedded in an ongoing statewide maternity care evaluation project led by an NGO partner in New York state. The survey was administered online from March 2016 to June 2017. To ensure ample inclusion of historically marginalized groups the survey was extended for a longer time frame for this group.

### Inclusion criteria

In the present analysis, we included respondents who answered questions about place of birth and ultimately gave birth in their planned setting (n = 1771). To reduce confounding, individuals who gave birth in a different setting than planned or were transferred (i.e. from a home or a birth center to a hospital) were excluded, as they report higher rates of disrespect and mistreatment than those with planned hospital births [31].

### Exposure variable

Three categories of birth setting, and intrapartum provider type were created for analysis: midwife in community setting (MW + comm), midwife in hospital setting (MW + hospital), and physician in hospital setting (MD + hospital). Community births attended by midwives included both births at home and births at a freestanding birth center, while hospital births included births at a hospital intrapartum unit or an in-hospital birth center.

### Outcome variables

Experiential outcomes were measured in four domains: (1) communication and decision-making autonomy, (2) respect, (3) mistreatment, and (4) time spent during visits.

Experiences with patient-provider communication and decision-making were assessed using the Mothers Autonomy in Decision-Making (MADM) scale, a 7-item instrument measuring the agency and autonomy a person experiences when participating in decision-making with a maternity care provider. This scale documents responses on six-point agree/disagree Likert scales, (scores ranging from 7 to 42) and was assessed for validity and reliability [34]. Scores from the MADM were used to create three categories. Individuals scoring in the bottom 33rd percentile were categorized as having low autonomy, while scores from the 34th through 65th percentiles indicated moderate autonomy, and scores in the 66th percentile and above indicated high autonomy. These cut offs enable clear delineation of low and high scores, with scores in the upper third constituting the optimum experiential outcomes. Respectful care was measured using the validated 14-item Mothers on Respect index (MORi), which assesses the nature of patient-provider interactions, and their impact on one’s sense of respect during maternity care [35]. Using a six-point Likert scale, participants rate their level of comfort when discussing care with their maternity care provider, the impact of provider interactions on their willingness to ask questions, and perceptions of racism or discrimination when receiving care. Scores from the MORi were collapsed into categories of low, medium, and high respect using the same approach as for the MADM scale.

Mistreatment was measured using the Mistreatment Index (MIST), a set of patient designed indicators of mistreatment, abuse, neglect, and health human rights violations [31]. Participants reported whether (yes/no) they experienced: “personal information shared without consent, violation of physical privacy, being shouted at or scolded by provider, provider threatened to withhold treatment or force acceptance of a treatment, provider threatened you in any other way, provider ignored or refused request for help or failed to respond in a timely manner, and experience of physical abuse”. Items were collapsed into a dichotomous mistreatment variable to reflect *any* negative experience versus no negative experiences. Time spent during prenatal visits (‘I felt I had enough time during my prenatal visits’; 6-point agree/disagree Likert scale) was also included as literature on what matters to service users often includes time as a critical factor in building trusting care relationships [36, 37]. The “had enough time” variable was collapsed into yes (strongly agree/agree) or somewhat/no (somewhat agree/somewhat disagree/disagree/strongly disagree) to compute the dichotomous variable.

### Covariates

Sociodemographic covariates included race, number of previous births, maternal age at last birth, nativity, highest level of education, total household income before taxes, source of payment for care, and whether respondents experienced elevated pregnancy risk. Since social risk factors may impact access to different models of maternity care and quality of care, while also being proxies for systemic inequities, we created five composite measures of social risk reported during pregnancy or in the year leading up to it by grouping variables under five categories. These included (1) primary care concerns (My children’s health, access to women’s health services, lack of health insurance) (2) mental health or substance use concerns (peace of mind/stress/mental health, depression, counseling for mental health, treatment for depression, problems with drug dependency, daily alcohol use, drug/alcohol treatment program, smoking (tobacco) and help to quit smoking; (3) financial and food concerns (access to healthy food, Inability to find work, inability to meet financial obligations, heat or electricity turned off, inability to buy enough food, during your recent pregnancy, need for food subsidies, state sponsored health plan, Temporary Assistance for Needy Families (TANF), assistance from Indian Health Services, Public child-care subsidies; (4) housing concerns (housing subsidies or assistance, access to safe housing, housing instability), and (5) safety concerns (Violence against family or community and violence in neighborhood, intimate partner violence, police violence, yourself or someone in your family, safe house or shelter from abuse (See Appendix for specific factors captured in each social risk factor category). Creating these 5 categories also allowed us to offer further dimension to our analysis which would have been otherwise unavailable due to small cell sizes for individual questions.

### Analysis

We computed descriptive statistics for the sample. We conducted chi-square analyses and independent t-tests in STATA Statistics version 17 to assess differences between the three provider types and setting exposure groups across socio-demographic and clinical characteristics and self-reported experiences of care. Outcomes were dichotomized in order to calculate the odds of different care experiences. Using bivariate logistic regression, the odds of experiencing each outcome were estimated to compare community-based midwifery and hospital-based midwifery care with hospital-based physician care (reference group). Outcome variables with statistically significant relationships were included in multivariate logistic regression models adjusted for socio-demographic and clinical characteristics as well as social risk factors that differed significantly between the three exposure groups. The significance level for all analyses was set a priori at 0.05. To further elucidate the impact of setting on experiences of care, we ran a secondary logistic regression analysis comparing midwives providing care in community settings (home and freestanding birth centers) to midwives providing care in hospital-based settings (reference group) for four primary outcomes: autonomy (MADM), respect (MORi), mistreatment (MIST), and time spent.

## Results

Of 2138 participants who completed all items on the GVtM survey, 1290 respondents met the selection criteria for this analysis and represented all 50 states. Participant demographics are displayed in Table 1. The GVtM study purposefully recruited individuals choosing to birth at home or in a freestanding birth center, therefore greater than half (55.4%) were cared for by midwives in community settings. Of those giving birth with midwifery care in community settings—a higher proportion identified as white, multiparous, held an Associate or college degree, were privately insured, and reported no elevated pregnancy risk. There were no significant differences in US nativity and household income between the groups. For factors in the social risk category, results varied across variables. A significantly lower proportion of participants receiving midwifery care in hospital settings reported primary care and housing concerns. However, there were no significant differences in health/substance use or concerns related to food & finances or safety. Additionally, a higher proportion of participants receiving physician care in hospital settings reported concerns related to mental health/substance use, housing, and safety concerns compared to other groups.

**Table 1:** Characteristics of participants, by provider and place of birth (n = 1771)

	MW + comm n = 980(55.3%)	MW + hosp. n = 275 (15.5%)	MD + hosp. n = 516 (29.1%)	p-value
Maternal race				
Asian	41 (4.2)	12 (4.4)	31 (6.0)	< 0.001
Black	88 (9.0)	50 (18.2)	129 (25.1)	
Latina/x	79 (8.1)	14 (5.1)	58 (11.3)	
Indigenous	31 (3.2)	8 (2.9)	11 (2.1)	
White	740 (75.6)	190 (69.3)	285 (55.4)	
Number of previous births				
0	0	0	2 (0.4)	< 0.001
1	287 (28.4)	125 (44.5)	239 (45.4)	
2–3	575 (57.0)	138 (49.1)	233 (44.2)	
4 or more	147 (14.6)	18 (6.4)	53 (10.1)	
Maternal age at last birth				
17–24	43 (4.5)	12 (4.5)	38 (7.8)	0.009
25–30	307 (31.9)	88 (33)	156 (31.9)	
31–39	548 (56.9)	160 (59.9)	256 (52.4)	
40 +	65 (6.7)	7 (2.6)	39 (8.0)	
Born in U.S.				
Yes	875 (89.6)	252 (91.0)	443(89.9)	0.789
No	102 (10.4)	25 (9.0)	50 (10.1)	
Highest level of education				
Less than college degree	203 (21.1)	38 (13.9)	98 (20.2)	< 0.001
College or Associate degree	482 (50.1)	121 (44.2)	215 (44.2)	
Graduate degree or more	278 (28.9)	115 (42.0)	173 (35.6)	
Main source of payment for maternity care				
Medicaid/CHIP	107 (10.9)	42 (15.1)	97 (19.4)	< 0.001
Private insurance	429 (43.9)	217 (77.8)	356 (71.3)	
Out of pocket	360 (36.8)	4 (1.4)	10 (2.0)	
Other	82 (8.4)	16 (5.7)	36 (7.2)	
Total household income before taxes				
$0–19,999	57 (6.0)	5 (1.9)	27 (5.6)	0.059
$20,000–49,999	214 (22.6)	55 (21.2)	106 (22.2)	
$50,000–99,999	344 (36.3)	85 (32.8)	164 (34.3)	
$100,000–159,999	192 (20.3)	74 (28.6)	116 (24.3)	
$160,000 or more	140 (14.8)	40 (15.4)	65 (13.6)	
Elevated pregnancy risk	93 (9.2)	45 (16.0)	203 (38.3)	< 0.001
Social risk factors				
Primary care concerns	470 (46.6)	111 (39.5)	217 (40.9)	0.03
Mental health/ substance use	468 (46.4)	132 (47.0)	263 (49.6)	0.475
Financial and food concerns	446 (44.2)	104 (37.0)	227 (42.8)	0.098
Housing concerns	71 (7.0)	13 (4.6)	55 (10.4)	0.008
Safety concerns	202 (20.0)	44 (15.7)	114 (21.5)	0.132

MW + comm (Midwifery care in community setting); MW + hospital (midwifery care in hospital setting); MD + hospital (physician care in hospital setting)’

*Chi-square; alpha = 0.05

The bivariate analyses findings reported in Table 2 show that the provider + setting groups differed significantly across all domains: communication and decision-making autonomy, respectful care, mistreatment, and time with healthcare provider. People cared for by midwives in community settings reported significantly higher autonomy, higher respect, lower incidence of mistreatment, and reported spending enough time with their midwife providers compared to both those who were cared for by midwives in hospital settings and those cared for by physicians in hospital settings. People receiving care from physicians in hospitals and midwives in hospitals reported far more instances of mistreatment compared to those receiving care from midwives in community settings.

**Table 2:** All experiential indicators by provider type and place of birth

	MW + comm n (%)	MW + hospital n (%)	MD + hospital n (%)
Communication and decision-making autonomy			
Mothers autonomy in decision making (MADM) (p < 0.001)			
High autonomy	478 (51.3)	64 (24.2)	78 (15.8)
Low and medium autonomy	454 (48.7)	201 (75.8)	416 (84.2)
Respectful care			
Mothers on Respect Index (MORi) (p < 0.001)			
High respect	493 (54.5)	47 (18.4)	74 (15.7)
Low and medium respect	412 (45.5)	209 (81.6)	396 (84.3)
Mistreatment			
Experienced any mistreatment: (p < 0.001)			
Any Mistreatment	44 (4.6)	56 (21.0)	141 (28.2)
No Mistreatment	904 (95.5)	211 (79.0)	359 (71.8)
Time with health care provider			
Had enough time during prenatal visits (p < 0.001)			
Yes	912 (96.2)	217 (81.3)	334 (66.8)
Somewhat or no	36 (3.8)	50 (18.7)	166 (33.2)

MW + comm (Midwifery care in community setting); MW + hospital (midwifery care in hospital setting); MD + hospital (physician care in hospital setting) MADM -Mothers on Autonomy in Decision Making Scale [34], Mothers on Respect Index [35] Mistreatment Index [31])

Unadjusted and adjusted odds ratios for experiential outcomes by the combined provider-setting groups are shown in Table 3. After adjusting for sociodemographic characteristics, clinical factors, and social risk factors, compared to those participants who received physician care in hospital settings (reference group), people receiving midwifery care in community settings had more than five times the odds of experiencing high autonomy (aOR:5.22, 95%CI: 3.65–7.45) and high levels of respect (aOR = 5.39, 95%CI: 3.72–7.82) and 14.65 times the odds of having enough time during prenatal visits (95%CI: 8.41–25.51). People receiving midwifery care in community settings also had significantly lower odds of reporting mistreatment compared to those receiving physician care in hospital settings (aOR = 0.16, 95% CI: 0.10–0.26).

**Table 3:** Unadjusted and adjusted odds of autonomy, respect, mistreatment, and time spent by provider type/place of birth dyad

	Unadjusted odds ratio (95% CI)	Adjusted odds ratio^a^ (95% CI)
High autonomy (MADM)		
MW + Comm	5.62* (4.27–7.38)	5.22** (3.65–7.45)
MW + hospital	1.70* (1.17–2.46)	1.70* (1.11–2.61)
MD + hospital	Ref.	Ref.
High respect (MOR)		
MW + Comm	6.40** (4.84–8.48)	5.39** (3.72–7.82)
MW + hospital	1.20 (0.80–1.80)	1.32 (0.83–2.09)
MD + hospital	Ref.	Ref.
Experienced at least one form of mistreatment (MIST)		
MW + Comm	0.12** (0.09–0.18)	0.16** (0.10–0.26)
MW + hospital	0.68* (0.47–0.96)	0.77 (0.50–1.17)
MD + hospital	Ref.	Ref.
Had enough time during prenatal visits		
MW + Comm	12.59 ** (8.60–18.44)	14.65 ** (8.41–25.51)
MW + hospital	2.16** (1.51–3.09)	1.95* (1.28–2.97)
MD + hospital	Ref.	Ref.

^a^Model adjusts for race, number of previous births, maternal age at last birth, highest level of education, main source of payment for maternity care, elevated pregnancy risk, BMI, primary care concerns, and housing concerns. **p < 0.001; *p < 0.05

When comparing people cared for by midwives in hospital settings to those cared for by physicians in hospitals, there were statistically significantly higher odds of reporting high autonomy (aOR = 1.70, 95% CI: 1.11–2.61) and more time spent with provider (aOR = 1.95, 95% CI: 1.28–2.97) but no differences in reported experiences of respect, and mistreatment after adjusting for covariates.

*Finally,* to better understand differences observed between midwife providers by setting, we compared the reported experiences of those who received midwifery care in a community setting to those who received midwifery care in the hospital setting (Table 4). In this analysis, since this regression model compares midwifery care across the two settings, (excluding the observations for physicians) there were 1255 eligible respondents.

**Table 4:** Crude and adjusted odds of high scores for MADM, MOR, & MIST for people cared for by midwives in community settings, compared to midwives in hospital settings (n = 1255)

	OR (95% CI)	aOR^a^ (95% CI)
High Autonomy (MADM)	3.32** (2.45–4.51)	2.97** (2.66–4.27)
High respect (MOR)	5.34** (3.81–7.49)	4.15** (2.81–6.14)
Experienced at least one form of mistreatment (MIST)	0.18** (0.12–0.28)	0.20** (0.11–0.34)
Had enough time during prenatal visits	5.83** (3.73–9.11)	8.06** (4.26–15.28)

^a^Model adjusts for race, number of previous births, maternal age at last birth, highest level of education, main source of payment for maternity care, elevated pregnancy risk, BMI, primary care concerns, and housing concerns. **p < 0.001; *p < 0.05

In unadjusted models, people cared for by midwives in community settings had significantly higher odds of reporting high autonomy (OR 3.32, 95% CI: 2.45–4.51) and high respect (OR 5.34, 95% CI: 3.81–7.49), and lower odds of reporting any mistreatment (OR 0.18, 95% CI: 0.12–0.28), compared midwives in hospital settings. In the adjusted model, people cared for by midwives in community settings had significantly higher odds of reporting high levels of autonomy (aOR: 2.97, 95% CI: 2.66–4.27) and respect (aOR: 4.15, 95% CI: 2.81–6.14), had far lower odds of reporting any mistreatment (aOR: 0.20, 95%CI: 0.11–0.34), and reported significantly higher odds of having enough time during their care (aOR: 8.06, 95%CI: 4.26–15.28) compared to people cared for by midwives in hospital settings in the adjusted model.

## Discussion

While disparate outcomes for Black women and people and other systematically excluded groups are persistent and well documented, research on models of care that are more likely to lead to healthier and more desirable outcomes remains limited [38]. Our findings demonstrate clear evidence for how midwifery care as practiced outside of institutional settings offers childbearing people a greater likelihood of experiencing respect, autonomy, and satisfying engagement during one’s childbearing journey.

In our analysis, compared to people receiving care from a physician in a hospital, participants with midwifery care in community settings had more than five times the odds of reporting high levels of decision-making autonomy and were five times more likely to report that their provider showed them high levels of respect. Participants also reported fourteen times the odds of having enough time in prenatal visits with community midwives than when cared for by physicians. Participants receiving midwifery care in hospital settings were almost two times more likely to report having enough time during their prenatal visits. These results are consistent with previous studies demonstrating that independent models of midwifery-led care in homes and freestanding birth centers, which can encompass cultural and emotional aspects of care, and have sufficient time to provide relationship-based care, enhance the quality of care experiences and may contribute to a sense of personal safety [39, 40].

While maternity care in the U.S. is predominantly provided by obstetricians, growing discourse calls for the comprehensive values-based care offered by the midwifery care model as practiced across birth settings (home, freestanding birth center, and hospitals) [41]. The integration of midwifery care *across* settings would improve care quality and improve maternal health outcomes in the U.S. [1, 42]. Midwifery care and birth center care (overwhelmingly provided by midwives) are consistently identified as key strategies needed to enhance perinatal health outcomes [39, 43–45]. Yet, compared to countries that demonstrate healthier and safer outcomes, the organization of maternity care in the US provides limited access to choice in birth settings [including hospitals, freestanding birth centers (FBC), and homes] and limited integration of midwifery across maternity care services [22, 42, 46].

By measuring experiences of care stratified by both provider and setting, our study demonstrates the importance of the care setting in shaping patient outcomes and experiences. Partcipants cared for by midwives in community settings reported better care experiences than those cared for by physicians in the hospital, however, these benefits did not remain consistent for those cared for by midwives working in hospital settings. While participants cared for by midwives in hospitals reported more autonomy and more time spent in prenatal care compared to physicians, there were no differences in the levels of respectful care or mistreatment reported. This suggests that the setting where midwifery care is delivered has a significant impact on the capacity to operationalize key tenants of the midwifery model [47].

### Enabling midwifery care environments

Research conducted by Vedam et al. [22] and Yang et al. [23] demonstrated the potential for midwifery care to improve population level health outcomes if buttressed by structural policies that support autonomous midwifery practice, such as licensing, full scope of practice, access to midwifery care, fair insurance reimbursement and enhanced regulations. In states where midwives practiced with greater autonomy, Yang et al. [23] demonstrated lower odds of cesarean delivery, preterm birth, and low birth weight, compared with states with more limited midwifery autonomy. Vedam et al. [22] also provided strong evidence that in states where the regulatory environment for midwifery practice facilitated autonomous, full scope of practice, there were far better outcomes for women and childbearing people—including higher rates of spontaneous vaginal delivery, vaginal birth after cesarean (VBAC), and breastfeeding, and significantly lower rates of cesarean, preterm birth, low birth weight infants, and neonatal death. Both studies suggest that midwives practicing to the fullest expanse of their scope is critical to achieving optimum clinical outcomes. Subsequent investigations demonstrate the effect of midwifery care integration on experiential outcomes. Our findings support that there is a potent influence of the context for midwifery practice and enabling practice environments in institutional settings on experiential outcomes [27–29, 48].

Given the hierarchical structure and organization of maternity care in the US—as an obstetrician-led, hospital-centric, technocratic space, more robust methods are needed to also differentiate the impact of the models and settings of care, a deliberate move away from analysis by provider type alone. Our findings suggest that midwives providing their care outside of institutions—in homes and freestanding birth centers—are better able to support core principles of person-centered care rooted in a human rights approach which centers respect, relationship, and autonomy in decision-making. However, when the midwifery care model is within an institution such as a hospital, the constraints and culture of that environment are challenged to support the core values of midwifery [12, 49]. Global inclusion of person-centered care, which is organized to support a person’s autonomy, provide a respectful approach, and support for informed and culturally relevant decision-making is scarce in institutional settings [26, 50]. Our analysis of intragroup differences among midwives (community and hospital) demonstrates that the implementation of the midwifery care model is deeply influenced by the setting itself.

Our findings add to evidence showing the model itself seems to be strongly influenced by the setting in which care is given—with community settings (home and freestanding birth centers) offering greater likelihood of support and the hospital settings being limited by the constraints of a medical approach to care which deprioritizes experiential outcomes [51]. As presently enacted, hospital maternity settings do not provide an enabling environment for full realization of the midwifery model of care. An enabling environment is defined as the *social* system within which people function [52]. All the rules, laws, policies, power relations and social norms that govern engagement in these spaces are considered impactful to the overall functionality and culture of a system [53]. This confirms global understanding that the culture and organization of the care environment itself serves as a limiting or enabling factor in ensuring high quality care [48, 49]. Singular focus on provider behaviors fails to address the complex and intersecting factors that determine the quality of experiential care.

Challenges in operationalizing the midwifery model of care in hospitals are often due to the dominant physician-based practice model, which is pathology-focused and technocratically driven [54, 55]. The tendency for practitioners in hospitals to rely heavily on interventions creates a challenge to practice autonomy for both the service user and the midwife [12, 49, 56]. The confines of the practice policies, approaches to risk stratification and interprofessional dynamics are all factors that limit the midwifery model from flourishing in hospital and institutional settings [48, 57]. As Newnham and Kirkham argue “large institutions that prioritize a midwife–institution relationship over a midwife–woman relationship are in themselves unethical and inimical to the midwifery philosophy of care.” [58, p. 2147]. Our findings suggest that while ‘how’ care happens matters, ‘where’ that care happens is equally important.

### Implications for practice

Deep systemic and organizational changes are necessary to support the integration of midwifery across the range maternity care services. Two strategic directions and impact investments could enhance perinatal care services: (1) integration of a human rights-based framework across all care delivery settings and (2) restructuring hospital-based care to allow for more optimal midwifery care integration to build facility policies to enable the midwifery care model to thrive.

Maternal health care often happens without explicit commitment to a human rights-based approach to care. A limited framing of professional ethics as solely dependent on interpersonal interactions and biases, fails to consider the structural and institutional factors that also threaten human rights and dictate the social nature of human interactions [59, 60]. Our findings indicate a clear need to improve experiences of care in hospital settings and shift institutional approaches to better align with person-centered models of care. A pragmatic example of how healthcare praxis can incorporate rights-based principles is offered in the ‘Black Birthing Bill of Rights’ [61]. The resource visually outlines core principles of autonomy and respect in birth. It is also intended to provide guidance to hospitals, health care providers, government health agencies and others to “change/improve their ethic, policies, and delivery approach to serving Black women and persons throughout the birthing process” [61]. This is a clear and direct example of orienting healthcare practice and relationships to uphold the core principles of human dignity, autonomy and self-determination.

Given the prevalence of midwives working within the hospital setting—their philosophical approach to care can be difficult to actualize as institutional values are focused on profitability and risk profiling designed to locate pathology. These values ultimately drivs divestment from supporting physiologic labor and birth and the psychosocial components of care [49, 57]. Maternity care in the US is a medical model of care led by physicians which predominantly operates through a hierarchical approach over a collaborative approach [62]. For a person-centered care model to flourish in all birth settings, a horizontal approach that promotes active collaboration built on principles of professional regard, mutual respect, and trust in the expertise offered by each provider type is essential [63]. For example, care bundles with simulations solely focused on clinical management without consideration of person-centeredness and interprofessional collaboration perpetuate the gap in our ability to measure and address the psychosocial dynamics that impact outcomes. To address the comprehensive needs of women and childbearing people, healthcare quality improvement initiatives must incorporate affective and social dimensions of care as system level drivers of care—not only relegated to the responsibility of individual providers but embraced to orient the organization and implementation of care. Research continues to show that respect, autonomy and informed decision making are highly valued by perinatal care service users—both locally and globally [25, 64] and are key to ensuring anti-oppression, culturally respectful care becomes the new standard of care [25, 65]. Increased integration of midwifery care into all care pathways—at home, in freestanding birth centers and in hospitals, may ensure greater ability to offer high quality and culturally safe experiences of care to all childbearing people.

### Limitations

The sampling strategy for this study was not designed to be representative, limiting the generalizability of the findings. Participants of color were oversampled to explore factors associated with wide disparities in U.S. birth outcomes by race/ethnicity and the systematic exclusion of racialized populations from research. This survey also intended address the lack of data on experiences of childbearing care outside hospital settings. In previous studies, analyses stratified by both birth setting and provider were limited due to small sample sizes. Our sampling strategy allowed for rigorous comparisons by birth setting and provider type.

A large proportion of our sample reported living in New York state. However, the strength of findings even after controlling for variation in sociodemographic characteristics, and pregnancy risk factors suggest that differences in experiences of care based on setting are unlikely to be limited to a particular state or demographic but may represent a large-scale, systemic problem.

It is also possible that multiple participants gave birth in the same hospitals (especially for births in New York) and we were unable to control for clustering of births within hospitals. Hence, our analysis does not account for the well-documented variations in the quality of care between hospitals [66]. Additionally, we note that our analysis did not assess the potential mitigating effects of continuity of provider across the arc of prenatal care to labor and birth care on the care experience.

## Conclusion

Reports of disrespect, mistreatment and coercion continue to emerge while leading to ongoing mistrust and avoidance of healthcare [31, 67, 68]. While midwifery care offers an approach that is rooted in values such as trust, autonomy, personhood, and informed choice, little research contextualizes how quality of midwifery care may be linked to the setting in which midwives practice. The Giving Voice to Mothers-US study confirms that how midwifery care is delivered is limited by where midwifery care is delivered.

Growing discourse and critique of safety and quality in birth enhances understanding of how existing models of maternity care must also evolve. More precise consideration of the varied pathways through which care is provided and their influence on experiential outcomes offers a more complete perspective on where to direct energy and resources that directly center the needs and desires of childbearing people. Addressing the comprehensive needs of women and childbearing people requires an approach that allows for both the affective (respect, compassion, and kindness) and moral (justice and personal autonomy) dimensions of care to be actively designed, implemented, and protected in all settings, by all providers.

## Data Availability

Data is available upon request.
